# The effect of cannabidiol on seizure features and quality of life in drug-resistant frontal lobe epilepsy patients: a triple-blind controlled trial

**DOI:** 10.3389/fneur.2023.1143783

**Published:** 2023-07-03

**Authors:** Seyyed Reza Ebadi, Kiarash Saleki, Tanin Adl Parvar, Negin Rahimi, Vajiheh Aghamollaii, Sara Ranji, Abbas Tafakhori

**Affiliations:** ^1^Iranian Center of Neurological Research, Neuroscience Institute, Tehran University of Medical Sciences, Tehran, Iran; ^2^School of Management and Medical Education, Shahid Beheshti University of Medical Sciences, Tehran, Iran; ^3^Student Research Committee, Babol University of Medical Sciences, Babol, Iran; ^4^USERN Office, Babol University of Medical Sciences, Babol, Iran; ^5^Cognitive Neurology and Neuropsychiatry Division, Tehran University of Medical Sciences, Tehran, Iran; ^6^Department of Neurology, Roozbeh Psychiatric Hospital, School of Medicine, Tehran University of Medical Sciences, Tehran, Iran

**Keywords:** epilepsy, cannabidiol, drug-resistant seizures, neuropharmacology, neurology

## Abstract

**Background:**

Treatment-resistant epileptic seizures are associated with reduced quality of life (QoL). As polypharmacy with routine antiseizure medications has many side effects, novel add-on treatments are necessary. Recent research showed the efficacy of add-on therapy by cannabidiol (CBD) on refractory epilepsy. We attempted to extend data on the efficacy and safety profile of CBD in patients with frontal lobe treatment-resistant epilepsy.

**Methods:**

A total of 27 patients were recruited into two CBD (*n* = 12) and placebo (*n* = 15) groups. The CBD group received a highly purified liposomal preparation of the drug in addition to routine antiseizure medications. The placebo group only received antiseizure medications. This experiment followed a triple-blinding protocol. Outcome measures were seizure frequency, the Chalfont seizure severity scale (CSSS), and the quality of life questionnaire score (QOLIE-31) assessed at baseline, 4 weeks, and 8 weeks.

**Results:**

At 4 weeks, results indicated that a higher fraction of patients in the CBD group (66.67%) showed improvement in seizure, compared to the placebo group (20.00%). Before–after comparison revealed that CBD, unlike routine ADEs, was effective in reducing the occurrence of seizures at the study's final timepoint [mean difference 45.58, 95% CI (8.987 to 82.18), *p* = 0.009]. Seizure severity was not affected by study groups or time intervals (repeated-measures ANOVA *p* > 0.05). *Post-hoc* tests found that the QoLI-31 score was improved at 8 weeks compared to baseline [mean diff. −5.031, 95% CI (−9.729 to −0.3328), *p* = 0.032]. The difference in cases who experienced enhanced QoL was meaningful between the CBD and placebo groups at 8 weeks [RR: 2.160, 95% CI (1.148 to 4.741), *p* = 0.018] but not at 4 weeks (*p* = 0.653). A positive finding for QoL improvement was associated with a positive finding for seizure frequency reduction [*r* = 0.638, 95% CI (0.296 to 0.835), *p* = 0.001]. Interestingly, limiting the correlation analysis to cases receiving CBD indicated that QoL improvement was not linked with seizure parameters such as severity and frequency (*p* > 0.05).

**Conclusion:**

The present study suggests the benefit of a purified and highly efficient preparation of CBD for seizure frequency reduction and improvement of QoL in refractory frontal lobe epilepsy. Further study with longer follow-ups and larger sample size is advised.

**Clinical trial registration:**

https://www.irct.ir/trial/56790, identifier: IRCT20210608051515N1.

## Introduction

According to the World Health Organization (WHO), epilepsy is the most common chronic neurological disorder which affects over 50 million people around the globe (1). Focal epilepsy is the most prevalent subtype of adulthood epilepsy. Among focal epilepsy patients, temporal lobe epilepsy is the most common, followed by frontal lobe epilepsy (2). More than two-thirds of patients who have recently been diagnosed with epilepsy respond to therapy by antiseizure medications and go into remission without relapse for a long period. Despite all this and the introduction of various treatment methods, including anticonvulsant drugs, neuromodulation, surgery, and therapeutic interventions, the burden of refractory epilepsy continues to plague the remaining one-third of the patients (3).

Although the use of many antiseizure medications is associated with a reduction in the number of convulsive attacks in focal epilepsy and associated conditions, the information related to the effectiveness and safety of those drugs in drug-resistant conditions is limited. Indeed, there are 17 verified drugs for partial seizures (4–6). Safety, patient's tolerance, and pharmacoeconomic rationale would in such settings drive clinical therapeutic approaches in drug-resistant seizures (7). Data on add-on treatment are available for 13 antiseizure medications showing no clear distinction in terms of efficacy. Hence, testing novel herbal-based candidates is encouraged to overcome these obstacles. Overall, evidence on specific drug combinations remains rare and a combination of routine antiseizure medications with novel candidates such as herbal-based extracts such as cannabidiol (CBD) deserves further investigation (8).

The use of CBD for epilepsy has a long history. In fact, during the nineteenth century, British and American doctors reported cases of *cannabis indica* infusion being able to reduce seizures (9). Among the hundreds of phytocannabinoids present in the cannabis plant, the two forms of THC and CBD are the most widely studied. The use of combined products of different types of marijuana with a higher ratio of the active substance (CBD to THC) is increasingly favored in this era. These compounds have been effective in controlling convulsive attacks, especially in treatment-resistant epilepsy in children (10). The anticonvulsant effects of CBD have been proven in preclinical animal models (11). Many of the current available studies are done using Epidiolex drug (GW British company), which is the pure form of CBD in an oil-based solution drug form. The amount of the effective substance in this solution is 100 mg per milliliter of the drug solution. Moreover, other types of drugs with different pharmacokinetic mechanisms are being produced and developed in order to increase absorption, reduce the prescribed dose, and reduce the side effects of the drug (12). Furthermore, treatment-resistant epilepsy reduces the quality of life (QoL) which could be restored using CBD (13, 14). However, its ability to improve QoL in specific subtypes of refractory epilepsy, such as the frontal lobe subtype remains unclear. Resistant frontal lobe epilepsy responds very poorly to drugs and deserves further study. Challenges in electroclinical localization almost certainly are involved in worse outcomes in the surgical treatment of frontal lobe epilepsy in comparison with other epilepsy subtypes (15).

Resistant epilepsy may be associated with several conditions such as encephalopathic disorders, Dravet syndrome (16), Lennox–Gastaut syndrome (17), febrile infectious disease-related epilepsy syndrome (FIRES) (18), and tuberous sclerosis complex which are considered as one of the most resistant types of epilepsy (19). Worthy of note is that, in previous studies, most of the cases of treatment-resistant epilepsy were mixed or specific epilepsy syndromes (20). Therefore, checking the effectiveness, safety, and adjusting the appropriate dose of treatment in patients with specific and other common epilepsy syndromes such as frontal, occipital, or parietal epilepsy and even with other etiological roots should be on the agenda (21).

Taking the above into account, in this clinical trial study, we studied the effectiveness and safety of CBD at the level of pharmaceutical grade compound, which is 99.95% pure CBD (THC-Free and in liposomal form for which an absorbed drug during previous pharmacological studies is increased 2–3.4 times, as produced by KMT Company). Precisely, we explored seizure frequency and severity as well as QoL improvement in a population of patients with frontal lobe epilepsy resistant to drug treatments in a triple-blind randomized clinical trial. We test the hypothesis that CBD may enhance seizure parameters and/or quality of life in patients with drug-resistant frontal lobe epilepsy.

## Methods and materials

### Patient selection criteria

**Figure 1:**
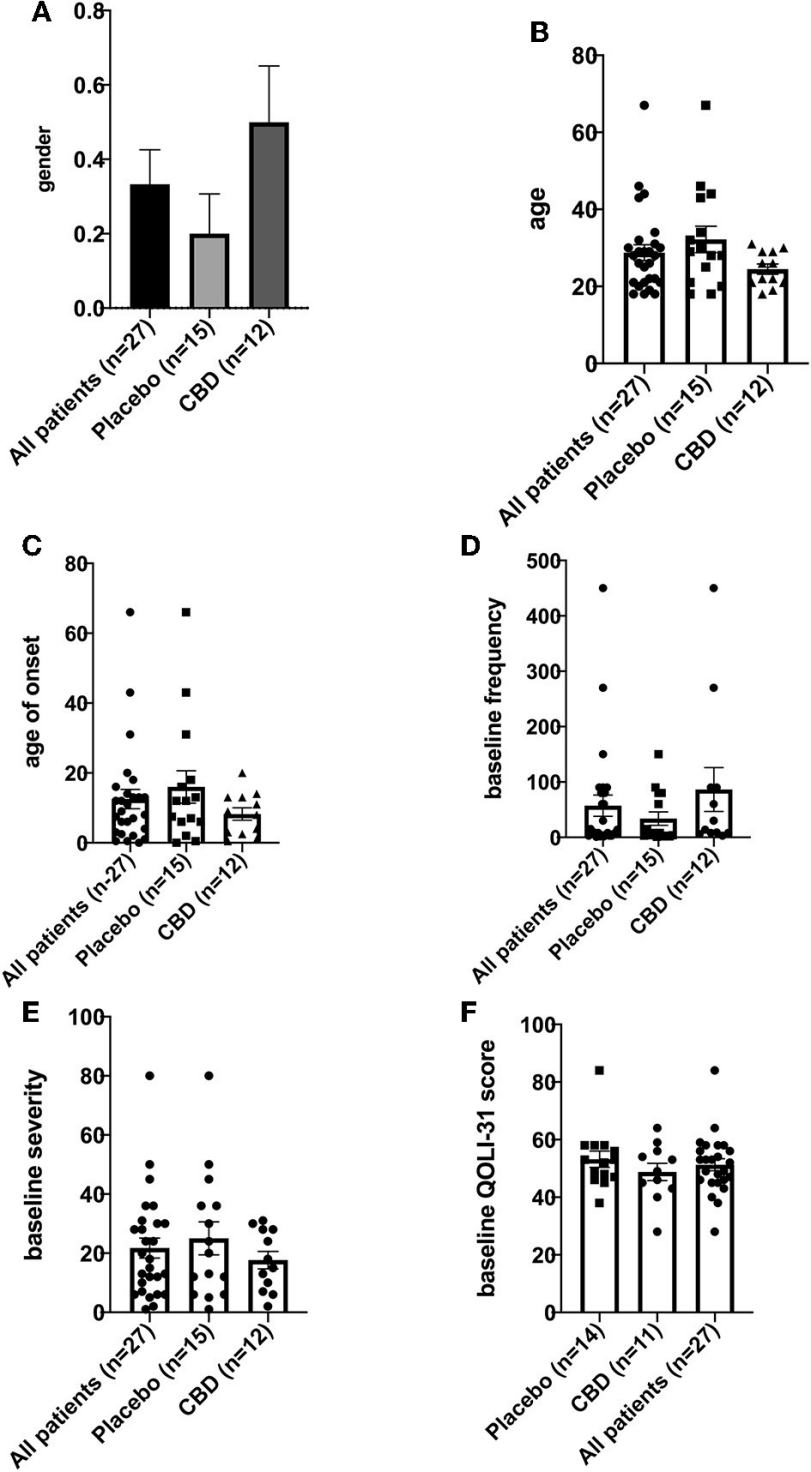
**(A–F)** Population demographics and baseline characteristics.

This experiment followed a triple-blind randomized clinical trial design. In this study, we enrolled 27 drug-resistant frontal lobe epilepsy adult patients (≥18 years old) of both genders from an epilepsy center of Imam Khomeini Hospital, Tehran, Iran. Simple randomization was performed by computer-generated numbers. Selected patients underwent long-term monitoring for seizures and showed resistance to at least two antiseizure medications. The diagnosis of frontal lobe epilepsy was confirmed for all patients based on clinical and paraclinical characteristics. Patients' IQs (who could complete QoL) ranged from 72 to 100 in this study. Baseline characteristics and patient demographics are provided in Table 1 and Figure 1.

**Table 1:** Population demographics and baseline characteristics.

**Group**	**Total (*n* = 27)**	**Placebo (*n* = 15)**	**Cannabidiol (*n* = 12)**	***p-*value^a^**
Gender (male/female)	9/18 (33.33%/66.66%)	3/12 (20.00%/80.00%)	6/6 (50.00%/50.00%)	0.100
Age (mean, SEM)	28.78, 2.08	32.20, 3.40	24.50, 1.31	0.154
Age of seizure onset (mean, SEM)	12.56, 2.76	16.00, 4.65	8.25, 1.77	0.462
**Baseline characteristics**				
Seizure frequency	57.33, 19.07	34.00, 11.97	86.50, 39.58	0.115
Seizure severity	21.78, 3.38	25.07, 5.57	17.67, 2.97	0.254
QoLI-31	51.28, 2.07	53.21, 2.83	48.82, 3.01	0.491

Two-sided p-values are presented for the placebo compared to the cannabidiol group. Appropriate Welch's t-test/non-parametric Mann–Whitney test and chi-square tests are performed. ^a^corresponds to the table caption (about t-tests and chi-square etc).

The inclusion criteria were the diagnosis of focal frontal lobe refractory epilepsy (lack of response or insufficient response to treatment with two or more common antiseizure medications) with an electroencephalogram (EEG) compatible with frontal lobe epilepsy and at least monthly seizures. The exclusion criteria were positive history of using marijuana or hashish compounds in the last month, pregnancy, concomitant non-epileptic seizures, and use of any of the following drugs such as clobazam, desmethylclobazam, eslicarbazepine, topiramate, zonisamide, and warfarin. This was because the interaction of some of these medications with CBD was significant (22, 23). Patients with cognitive problems were excluded from quality of life analysis if their mental problems prevented them from completing this assessment.

This study was approved by the ethics committee of the Tehran University of Medical Sciences, Iran (Ethics approval code: IR.TUMS.MEDICINE.REC.1400.130). The protocol for the conduct of this experiment was registered with the Iran Registry of Clinical Trials (IRCT) with the registration code IRCT20210608051515N1.

### Treatment protocols

The participants were assigned to intervention (CBD) and placebo groups while continuing their usual antiseizure treatment, which may include phenobarbital, valproic acid, acetazolamide, levetiracetam, carbamazepine, lamotrigine, lacosamide, gabapentin, primidone, clonazepam, phenytoin, and oxcarbazepine. Each group received a specific dose of the medication of the drug prepared by KMT company (KMT, Iran; https://www.kmtmed.com/) containing cannabidiol (INOVO Lipomed Liposomal CBD, KMT) or placebo. Patients in the CBD group were administered CBD in liquid form containing 40 mg/ml of the active substance (CBD) with appropriate levels of preservatives and flavorings based on the protocol prepared by KMT Pharmaceutics. It was prescribed 70 mg (equivalent to 1.75 ml) in the 1st week, then 140 mg (equivalent to 3.5 ml) in the 2nd week, and 210 mg (equivalent to 5.25 ml) in the 3rd to final week as a single dose.

During the baseline period, patients were given the first dose of their medicine and underwent history-taking (e.g., the number of seizures per month), neurological examination before the start of the study, and the quality of life in epilepsy, 31 questions (QOLIE-31, valid and reliable in the Persian language of QOLIE-89 questionnaire) assessment (24) and the Chalfont Seizure Severity Scale (CSSS) (25, 26) were used. Patients' contact information was collected, and they were provided with recommendations on taking their drug by gradually increasing the dose of the drug depending on the patient's tolerance. During the treatment period, included patients continued their previous antiseizure medications with the same fixed dose as before.

### Assessment of outcomes

Patients of both groups were followed-up for 8 weeks after receiving the first dose, and follow-up calls were made every 4 weeks (three timepoints). Cases of withdrawal from the study by the patient, side effects and their types, the frequency of seizures, and the type of complaints were recorded. After the completion of data collection, data analysis, and interpretation were done comparatively in baseline cases with follow-up by people who were blocked from the information of the patients of both groups and disease conditions (blinding of the analysis). During this statistical evaluation, the variables such as the mean percentage of reduction of convulsive attacks, percentage of freedom from convulsions, percentage of side effects by mentioning the types of occurrences, the statistical significance of the difference in the results of QOLIE-31 tests, the severity of seizures, and the drug side effects between two timepoints, baseline and follow-up, were evaluated.

Compliance with antiseizure medications and CBD was ascertained following routine clinical practice by asking patients and/or their caregivers. However, the level of drugs in the serum or pill-counting was not performed. Moreover, adverse events were recorded at the baseline, weeks 4, and 8 weeks.

### Blinding settings

All participants were blinded to their group assignment (drug or placebo) although they were informed that they will participate in a study in which they will take either drug or placebo options. Moreover, the label and specifications on the medicine bottles were similar for the drug and placebo. The color, taste, smell, and contents of the boxes were not distinguishable. The caregiver, the scientist, and the physician responsible for the diagnosis and treatment were all blinded to the therapy. Moreover, outcome assessment and statistical analysis of the data were performed without information about the grouping of participants. The safety and data monitoring committee was not aware of the details of participants who received the drug or placebo and their identity.

### Statistical analysis

Results were analyzed using version PRISM 8.4.3 (GraphPad Inc, USA) and Statistical Packages for Social Sciences (SPSS) version 26.0.0. The patient characteristic outcomes (age, age of seizure onset, gender, seizure severity, seizure frequency per month, and quality of life) were analyzed. The outcomes were compared between placebo and CBD groups, three timepoints in each group (repeated-measures ANOVA or mixed-effects to take into account missing values) and before-after comparison for each placebo and CBD. Optimal *post-hoc* analyses were performed for ANOVA analyses (e.g., Bonferroni, Tukey, or Šidák). The chi-squared test was used to compare gender (binary variable) between groups. Greenhouse–Geisser correction was performed when sphericity was violated (ε < 0.75). Descriptive statistics and continuous outcomes were reported as mean ± standard error of the mean (SEM). For normality testing, Shapiro–Wilk (*n* < 50) or other appropriate tests were used in combination with histogram analysis. Improvement of patients was analyzed as a binary outcome by the chi-square test or Fisher's exact test. The latter was utilized when the expected value is < 5. The statistical significance threshold was considered at a *p*-value of < 0.05.

## Results

### Patient demographics and baseline analysis

A total of 27 patients were enrolled; 15 (three male/12 female, ratio 0.25) were assigned to the placebo group and 12 (six male/six female, ratio 1) were assigned to the CBD group. The difference in gender between groups was not significant (*p* > 0.05). Age (mean, SEM) was 32.20, 3.40, and 24.50, 1.31 for placebo and CBD groups, respectively. Age of seizure onset (mean, SEM) was 16.00, 4.65, and 8.25, 1.77 for placebo and CBD groups, respectively. Results for comparison of placebo to CBD patients showed seizure frequency, seizure severity, and QoLI-31, which were not significantly different between groups (*p* > 0.05). For two patients, cognitive problems prevented the baseline evaluation of QoLI-31. One of these patients was in the placebo group and the other was in the CBD group.

### Seizure frequency

For the placebo group, mean (SEM) seizure frequency was 34.00 (11.97), 26.87 (11.38), and 24.67 (9.94) at baseline, week 4, and week 8, respectively. For the CBD group, seizure frequency was 86.50 (39.58), 55.42 (21.49), and 40.92 (15.09) at mentioned timepoints, respectively.

Repeated measures of two-way ANOVA analysis were performed for seizure frequency per month in the CBD and placebo patients at the baseline, week 4, and week 8. Results showed that the effect of timepoint at all values of receiving CBD or placebo (interaction of timepoint and CBD/placebo) was not significant (*p* = 0.187). Interaction accounted for 1.103% of the variance. After adjusting for matching, grouping (receiving CBD/placebo) accounted for 5.123% of the total variance (*p* = 0.204). Timepoint (baseline/week-4/week-8) accounted for 2.573% of the total variance, which was significant (*p* = 0.023). Moreover, *post-hoc* analysis showed that seizure frequency was significantly reduced at 8 weeks compared to the baseline [mean difference 27.46, 95% CI (2.909 to 52.01), *p* = 0.023]. Interestingly, seizure frequency was significantly decreased in the CBD group at 8 weeks compared to the baseline [mean difference 45.58, 95% CI (8.987 to 82.18), *p* = 0.009] but not in the placebo group (*p* > 0.999). This overall before-after comparison indicates that CBD, unlike routine ADEs, was effective in reducing the occurrence of seizures at the experiment's final timepoint. Seizure frequencies were not different between the CBD and placebo groups at all three studied timepoints (*p* > 0.05; Table 2).

**Table 2:** Multiple comparisons of seizure frequency.

**Source of variation**	***P*-value**	**Significance**	**Variation (percentage)**	***F*_(DFn, DFd)_**
Time x receiving CBD or placebo	0.1877	ns	1.103	*F*_(2, 50)_ = 1.730
Time	0.0237	^*^	2.573	*F*_(2, 50)_ = 4.035
Receiving CBD or placebo	0.2049	ns	5.123	*F*_(1, 25)_ = 1.695
Subject	< 0.0001	^****^	75.59	*F*_(25, 50)_ = 9.481
***Post-hoc*** **tests**				
**Multiple comparisons test**	**Adjusted** ***P*****-value**	**Significance**	**Mean difference**	**95.00% CI of diff**.
**(i) Placebo—cannabidiol**				
Baseline	0.1780	ns	−52.50	−119.6 to 14.64
Week 4	0.9032	ns	−28.55	−95.69 to 38.59
Week 8	>0.9999	ns	−16.25	−83.39 to 50.89
**(ii) Placebo**				
Baseline vs. week 4	ns	>0.9999	7.133	−25.60 to 39.87
Baseline vs. week 8	ns	>0.9999	9.333	−23.40 to 42.07
Week 4 vs. week 8	ns	>0.9999	2.200	−30.53 to 34.93
**(iii) Cannabidiol**				
Baseline vs. week 4	0.1213	ns	31.08	−5.513 to 67.68
Baseline vs. week 8	0.0099	^**^	45.58	8.987 to 82.18
Week 4 vs. week 8	0.9932	ns	14.50	−22.10 to 51.10
**(iv) Overall comparison of time points**				
Baseline vs. week 4	0.1786	ns	19.11	−5.441 to 43.66
Baseline vs. week 8	0.0235	^*^	27.46	2.909 to 52.01
Week 4 vs. week 8	>0.9999	ns	8.350	−16.20 to 32.90

Two-way ANOVA (repeated measures) was used due to which measurements for all participants were available. The ^*^, ^**^, ^***^, and ^****^ indicates the value of *p* < 0.05, *p* < 0.01, *p* < 0.001, and *p* < 0.0001 respectively.

The mean change in seizure frequency was −7.13 (4.81) and −31.08 (24.59) at 4 weeks and −9.33 (4.99) and −45.58 (26.34) at 8 weeks for placebo and CBD groups, respectively. This indicates that, at both timepoints, CBD provides a better reduction in seizure frequency. Repeated measures of two-way ANOVA were performed. Results showed timepoint x receiving CBD or placebo (interaction), timepoint, and receiving CBD or placebo were not significant sources of variation (*p* > 0.05). However, the subject was a significant source of variation (*p* < 0.0001). *Post-hoc* analyses found no difference between study groups at study timepoints (*p* > 0.05; Table 3).

**Table 3:** Seizure frequency change from the baseline at 4 and 8 weeks.

**Source of variation**	***P*-value**	***P*-value summary**	**Variation (percentage)**	***F*_(DFn, DFd)_**
Timepoint x receiving CBD or placebo	0.2058	Ns	0.2588	*F*_(1, 25)_ = 1.687
Timepoint	0.0900	Ns	0.4771	*F*_(1, 25)_ = 3.110
Receiving CBD or placebo	0.1996	Ns	6.199	*F*_(1, 25)_ = 1.736
Patient	< 0.0001	^****^	89.30	*F*_(25, 25)_ = 23.29
***Post-hoc*** **tests**				
**Multiple comparisons test**	**Adjusted** ***P*****-value**	**Significance**	**Mean difference**	**95.00% CI of diff**.
**(i) Week 4 change—week 8 change**				
Placebo	>0.9999	Ns	2.200	−12.85 to 17.25
Cannabidiol	0.1011	Ns	14.50	−2.331 to 31.33
**(ii) Placebo—cannabidiol**				
Week 4 change	0.6193	Ns	23.95	−29.97 to 77.87
Week 8 change	0.2532	Ns	36.25	−17.67 to 90.17

The ^*^, ^**^, ^***^, and ^****^ indicates the value of *p* < 0.05, *p* < 0.01, *p* < 0.001, and *p* < 0.0001 respectively.

**Figure 2:**
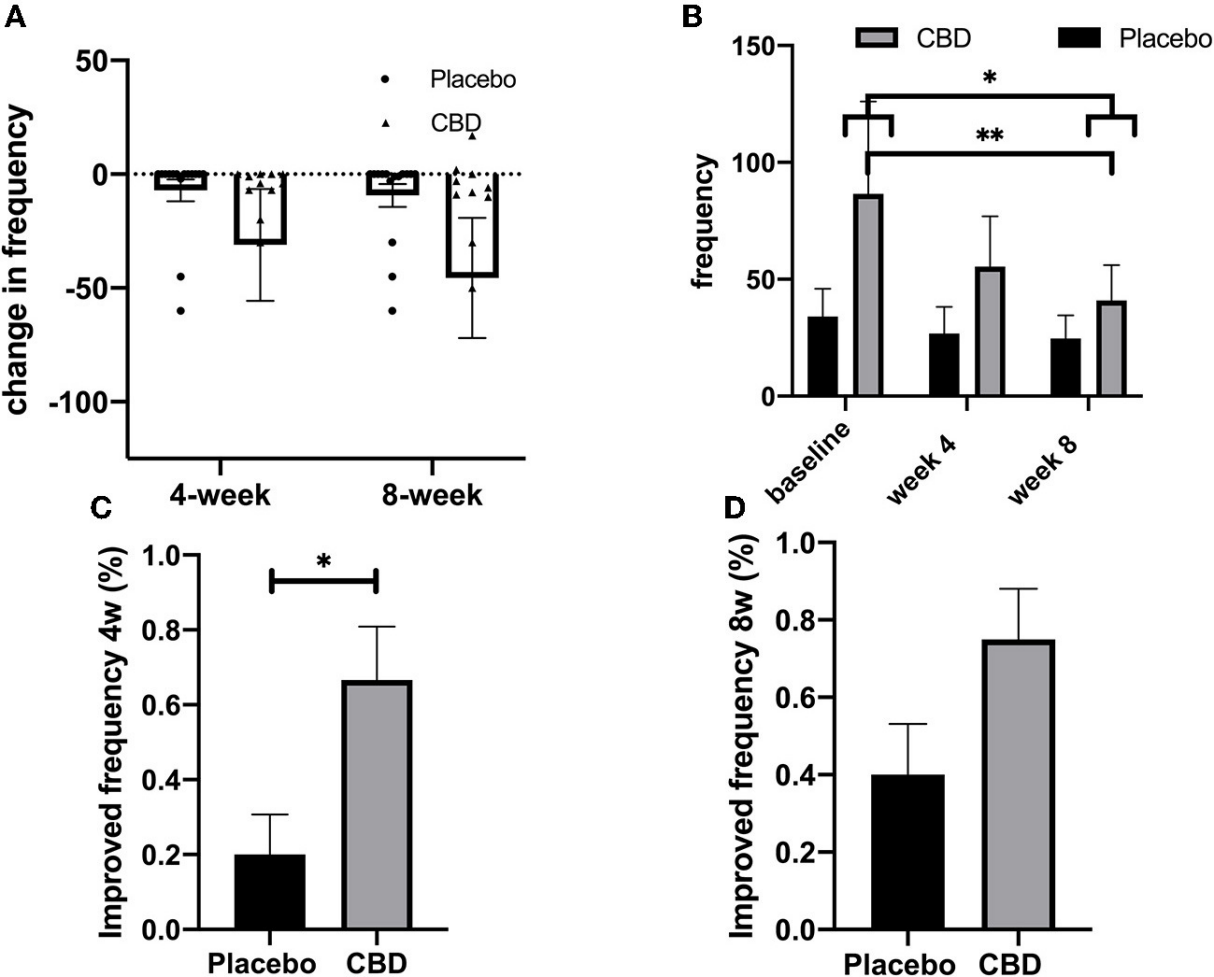
**(A–D)** Seizure frequency. The ^*^, ^**^, ^***^, and ^****^ indicates the value of *p* < 0.05, *p* < 0.01, *p* < 0.001, and *p* < 0.0001 respectively.

Additionally, the improvement of patients was analyzed as a binary outcome (improved/not improved). At 4 weeks, a higher fraction of patients in the CBD group (66.67%) showed significant improvement, compared to the placebo group (20.00%). The effect size for this difference was also relatively large [RR: 3.333, 96% CI (1.248 to 9.969), *p* = 0.014]. However, this comparison at 8 weeks was not significant [RR: 1.875, 95% CI (0.9509 to 3.975), *p* = 0.069]. The results of the seizure frequency analysis are shown in Figures 2A–D.

### Seizure severity

For the placebo group, the mean (SEM) seizure severity score was 25.07 (5.57), 17.93 (5.57), and 19.87 (5.50) at the baseline, week 4, and week 8, respectively. For the CBD group, seizure severity was 17.67 (2.97), 15.42 (3.03), and 13.58 (4.78) at mentioned timepoints. The lowest severity was found in the CBD group after 8 weeks.

Repeated measures of two-way ANOVA analysis were performed for seizure severity in the CBD and placebo groups at the baseline, week 4, and week 8. Results showed that the influence of timepoint at all values of receiving CBD or placebo (interaction of timepoint and CBD/placebo) was not significant (*p* = 0.402). The interaction was responsible for 0.3372% of the variance. Following adjustment for matching, grouping (receiving CBD/placebo) accounted for 2.253% of the total variance (*p* = 0.427). Timepoint (baseline/week-4/week-8) was responsible for 1.496% of the overall variation, which was statistically meaningful (*p* = 0.022). Additionally, *post-hoc* tests showed that seizure severity was highly attenuated at 4 weeks compared to the baseline [mean difference 4.692, 95% CI (0.03681 to 9.347), *p* = 0.047]. Seizure severity was significantly decreased in the placebo group at 4 weeks compared to the baseline (*p* = 0.019) but not at 8 weeks (*p* = 0.129). Following the Greenhouse–Geisser correction, however, this analysis was not significant (*p* > 0.05). Moreover, the direction of change in the point value of mean seizure severity increased from the 4th to the 8th week [mean diff. −1.933, 95% CI (−8.140 to 4.273), *p* > 0.999]. Seizure severity scores were not different between the CBD and placebo groups at all three recorded timepoints (*p* > 0.05). Overall, the results suggest that neither CBD nor routine therapy by antiseizure medications could effectively improve seizure severity (Table 4).

**Table 4:** Multiple comparisons of seizure severity.

**Source of variation**	***P*-value**	**Significance**	**Variation (percentage)**	***F*_(DFn, DFd)_**
Timepoint x receiving CBD or placebo	0.4024	ns	0.3372	*F*_(2, 50)_ = 0.9271
Timepoint	0.0222	^*^	1.496	*F*_(2, 50)_ = 4.112
Receiving CBD or placebo	0.4278	ns	2.253	*F*_(1, 25)_ = 0.6498
Patient	< 0.0001	^****^	86.69	*F*_(25, 50)_ = 19.06
***Post-hoc*** **tests**				
**Multiple comparisons test**	**Adjusted** ***P*****-value**	**Significance**	**Mean difference**	**95.00% CI of diff**.
**(i) Placebo—cannabidiol**				
Baseline	0.7637	ns	7.400	−9.036 to 23.84
Week 4	>0.9999	ns	2.517	−13.98 to 19.01
Week 8	>0.9999	ns	6.283	−12.40 to 24.97
**(ii) Placebo**				
Baseline vs. week 4	0.0192	^*^	7.133	0.9269 to 13.34
Baseline vs. week 8	0.1293	ns	5.200	−1.006 to 11.41
Week 4 vs. week 8	>0.9999	ns	−1.933	−8.140 to 4.273
**(iii) Cannabidiol**				
Baseline vs. week 4	>0.9999	ns	2.250	−4.689 to 9.189
Baseline vs. week 8	0.4535	ns	4.083	−2.856 to 11.02
Week 4 vs. week 8	>0.9999	ns	1.833	−5.106 to 8.772
**(iv) Overall comparison of time points**				
Baseline vs. week 4	0.0476	^*^	4.692	0.03681 to 9.347
Baseline vs. week 8	0.0509	ns	4.642	−0.01319 to 9.297
Week 4 vs. week 8	>0.9999	ns	−0.05000	−4.705 to 4.605

The ^*^, ^**^, ^***^, and ^****^ indicates the value of *p* < 0.05, *p* < 0.01, *p* < 0.001, and *p* < 0.0001 respectively.

The mean change in seizure severity score was −7.13 (3.32) and −2.25 (1.10) at 4 weeks and −5.20 (3.05) and −4.08 (2.71) at 8 weeks for placebo and CBD groups, respectively. The reduction in seizure severity was not significantly different between CBD and placebo. The two-way ANOVA test showed timepoint x receiving CBD or placebo (interaction), timepoint, and receiving CBD or placebo were not significant sources of variation (*p* > 0.05). Only subject was a significant origin of variation (*p* < 0.0001). *Post-hoc* testing detected no difference between different study groups at study timepoints (*p* > 0.05; Table 5).

**Table 5:** Seizure severity change from the baseline at 4 and 8 weeks.

**Source of variation**	***P*-value**	***P*-value summary**	**Variation (percentage)**	***F*_(DFn, DFd)_**
Timepoint x receiving CBD or placebo	0.2415	Ns	0.8464	*F*_(1, 25)_ = 1.440
Timepoint	0.9748	Ns	0.0005966	*F*_(1, 25)_ = 0.001015
Receiving CBD or placebo	0.4269	Ns	2.148	*F*_(1, 25)_ = 0.6525
Patient	< 0.0001	^****^	82.29	*F*_(25, 25)_ = 5.598
***Post-hoc*** **tests**				
**Multiple comparisons test**	**Adjusted** ***P*****-value**	**Significance**	**Mean difference**	**95.00% CI of diff**.
**(i) Week 4 change—week 8 change**				
Placebo	0.7289	Ns	−1.933	−6.924 to 3.057
Cannabidiol	0.8814	Ns	1.833	−3.747 to 7.413
**(ii) Placebo—cannabidiol**				
Week 4 change	0.4631	Ns	−4.883	−14.20 to 4.435
Week 8 change	>0.9999	Ns	−1.117	−10.43 to 8.201

The ^*^, ^**^, ^***^, and ^****^ indicates the value of *p* < 0.05, *p* < 0.01, *p* < 0.001, and *p* < 0.0001 respectively.

**Figure 3:**
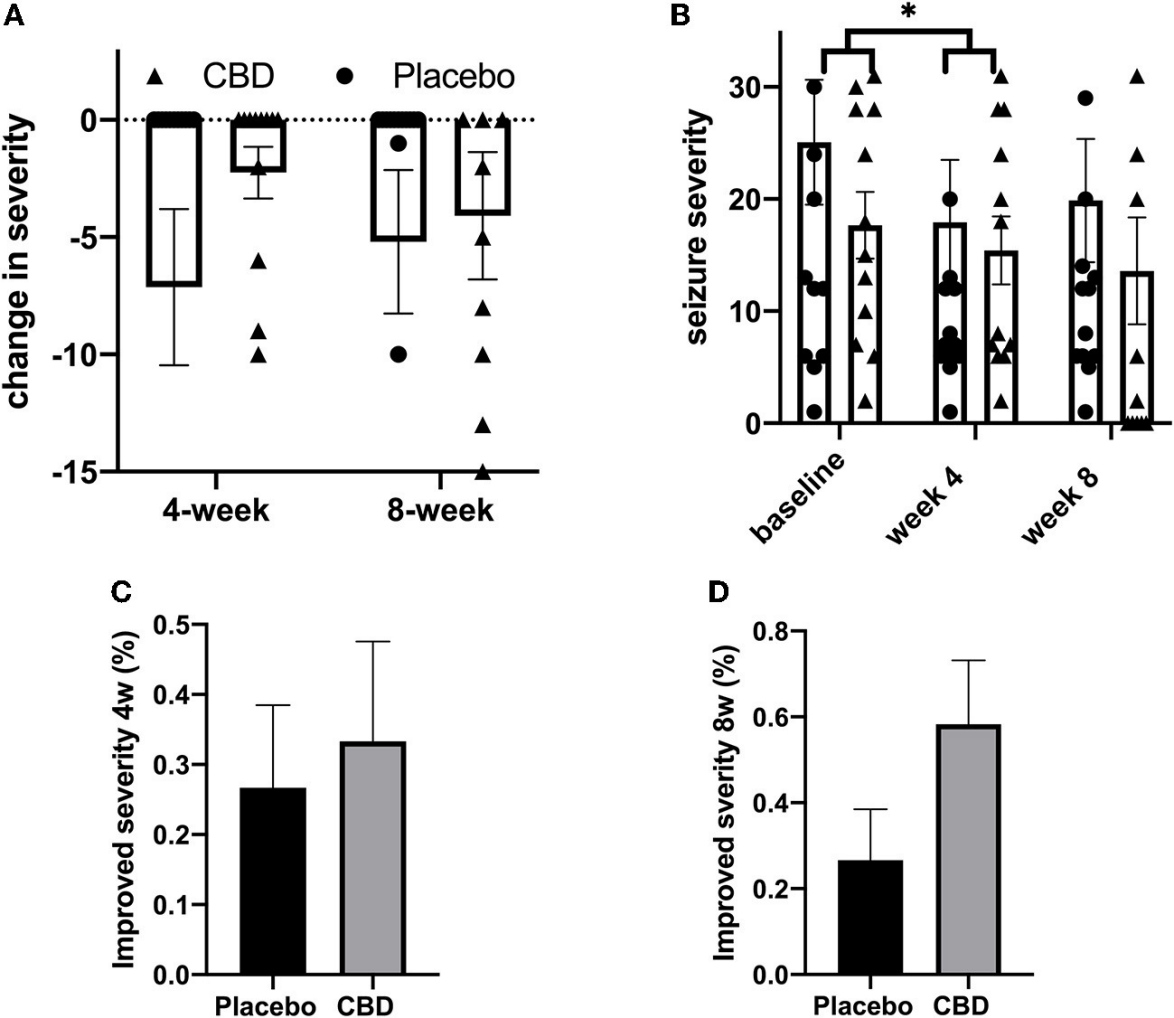
**(A–D)** Seizure severity analysis. The ^*^, ^**^, ^***^, and ^****^ indicates the value of *p* < 0.05, *p* < 0.01, *p* < 0.001, and *p* < 0.0001 respectively.

Seizure severity reduction was also analyzed as a binary parameter (improved/not improved). The severity reduction was not significantly different between the CBD and placebo groups at 4 weeks [RR: 2.188, 95% CI (0.8751 to 5.829), *p* = 0.096] and 8 weeks [RR: 1.250, 95% CI (0.4041 to 3.810), *p* = 0.0706]. Mean seizure severity scores for each patient throughout the study were lower in the CBD group but were not significantly different between CBD and placebo groups [*p* > 0.05, mean difference −5.400 (−18.48 to 7.675)]. Together, these data confirm that CBD and placebo are similarly ineffective in reducing seizures (Figures 3A–D).

### Quality of life

For QoL analysis, some patients with cognitive problems could not complete the evaluation (*n* = 2, one in each group). The other missing values were handled by the mixed-effects analysis.

For the placebo group, the mean (SEM) QoLI-31 score was 53.21 (2.83), 55.14 (2.99), and 55.67 (2.83) at the baseline, week-4, and week-8, respectively. For the CBD group, the seizure QoLI-31 score was 48.82 (3.01), 48.64 (4.26), and 58.90 (2.73) at mentioned timepoints, respectively. Similar to the previous results for seizure, the highest QoLI-31 score was found in the CBD group after 8 weeks.

Mixed-effects analysis was performed for QoL in the CBD and placebo groups at the baseline, week 4, and week 8. Results showed the influence of timepoint (*p* = 0.026) and timepoint at all values of receiving CBD or placebo (interaction of timepoint and CBD/placebo; *p* = 0.023). The significance of interaction shows that CBD across time could affect the QoLI-31 scores. The *post-hoc* test found that the QoLI-32 score was improved at 8 weeks compared to the baseline [mean diff. −5.031, 95% CI (−9.729 to −0.3328), *p* = 0.032]. For patients receiving CBD, the QoLI-31 score was significantly improved at week 8, compared to the baseline [mean diff. −9.140, 95% CI (−16.10 to −2.175), *p* = 0.006] and week 4 [mean diff. −9.321, 95% CI (−16.29 to −2.357), *p* = 0.005]. In summary, results indicate that CBD could improve QoL in combination with antiseizure medications. However, the antiseizure medication-only regimen could not improve QoL (Table 6A). Next, mentally healthy patients with a full evaluation of QoLI-31 at all three study timepoints were analyzed by two-way repeated measures ANOVA. Very similar results with the same levels of statistical significance were produced [time x receiving CBD or placebo (interaction) *p* = 0.01 and timepoint *p* = 0.02; Table 6B]. An additional three-way ANOVA was also performed with cognitive problems imported as a third factor into the analysis. Cognitive problems and their interactions with grouping and/or timepoint did not significantly contribute to the change in QoLI-31 (*p* > 0.05). Similar results were reported with grouping and timepoint interaction being a significant factor (*p* = 0.036; Table 6C).

**Table 6A:** Multiple comparisons of quality of life.

**Fixed effects (type III)**	***P*-value**	**Significance**	**Significant?**	***F*_(DFn, DFd)_**
Timepoints	0.0262	^*^	Yes	*F*_(2, 43)_ = 3.967
Receiving CBD or placebo	0.5564	ns	No	*F*_(1, 23)_ = 0.3563
Timepoints x receiving CBD or placebo	0.0236	^**^	Yes	*F*_(2, 43)_ = 4.092
***Post-hoc*** **tests**				
**Multiple comparisons test**	**Adjusted** ***P*****-value**	**Significance**	**Mean difference**	**95.00% CI of diff**.
**(i) Placebo—cannabidiol**				
Baseline	0.8973	ns	4.396	−6.310 to 15.10
Week 4	0.6800	ns	6.506	−7.173 to 20.19
Week 8	>0.9999	ns	−3.233	−13.51 to 7.048
**(ii) Placebo**				
Baseline vs. week 4	>0.9999	ns	−1.929	−7.896 to 4.039
Baseline vs. week 8	>0.9999	ns	−0.9219	−7.229 to 5.385
Week 4 vs. week 8	>0.9999	ns	1.007	−5.300 to 7.313
**(iii) Cannabidiol**				
Baseline vs. week 4	>0.9999	ns	0.1818	−6.551 to 6.914
Baseline vs. week 8	0.0064	^**^	−9.140	−16.10 to −2.175
Week 4 vs. week 8	0.0053	^**^	−9.321	−16.29 to −2.357
**(iv) Overall comparison of time points**				
Baseline vs. week 4	>0.9999	ns	−0.8734	−5.372 to 3.625
Baseline vs. week 8	0.0322	^*^	−5.031	−9.729 to −0.3328
Week 4 vs. week 8	0.0986	ns	−4.157	−8.855 to 0.5405

The ^*^, ^**^, ^***^, and ^****^ indicates the value of *p* < 0.05, *p* < 0.01, *p* < 0.001, and *p* < 0.0001 respectively.

**Table 6B:** Multiple comparisons of quality of life for patients with normal cognition.

**Fixed effects (type III)**	***P*-value**	**Significance**	**Variation (percentage)**	***F*_(DFn, DFd)_**
Time x receiving CBD or placebo	0.0139	^*^	4.777	*F*_(2, 40)_ = 4.763
Timepoint	0.0204	^*^	4.530	*F*_(1.820, 36.40)_ = 4.517
CBD or placebo	0.4301	Ns	2.241	*F*_(1, 20)_ = 0.6486
Patients	< 0.0001	^****^	69.10	*F*_(20, 40)_ = 6.889
***Post-hoc*** **tests**				
**Multiple comparisons test**	**Adjusted** ***P*****-value**	**Significance**	**Mean difference**	**95.00% CI of diff**.
**(i) Placebo—cannabidiol**				
Baseline	0.5987	Ns	5.900	−5.749 to 17.55
Week 4	0.5634	Ns	6.467	−6.232 to 19.16
Week 8	>0.9999	Ns	−3.233	−13.51 to 7.048
**(ii) Placebo**				
Baseline vs. week 4	>0.9999	Ns	−2.167	−8.110 to 3.777
Baseline vs. week 8	>0.9999	Ns	−0.6667	−6.610 to 5.277
Week 4 vs. week 8	>0.9999	Ns	1.500	−4.443 to 7.443
**(iii) Cannabidiol**				
Baseline vs. week 4	>0.9999	Ns	−1.600	−8.111 to 4.911
Baseline vs. week 8	0.0016	^**^	−9.800	−16.31 to −3.289
week 4 vs. week 8	0.0093	^**^	−8.200	−14.71 to −1.689
**(iv) Overall comparison of time points**				
Baseline vs. week 4	0.8761	Ns	−1.883	−6.291 to 2.524
Baseline vs. week 8	0.0152	^*^	−5.233	−9.641 to −0.8257
Week 4 vs. week 8	0.1943	Ns	−3.350	−7.758 to 1.058

The ^*^, ^**^, ^***^, and ^****^ indicates the value of *p* < 0.05, *p* < 0.01, *p* < 0.001, and *p* < 0.0001 respectively.

**Table 6C:** Three-way comparison of quality of life, mental status, and time.

**Fixed effects (type III)**	***P*-value**	**Significance**	**Significant?**	***F*_(DFn, DFd)_**
Timepoint	0.0991	Ns	No	*F*_(2, 39)_ = 2.454
(MR vs. no MR)	0.2960	Ns	No	*F*_(1, 21)_ = 1.149
(Placebo vs. CBD)	0.7458	Ns	No	*F*_(1, 21)_ = 0.1079
Timepoint x (MR vs. no MR)	0.6854	Ns	No	*F*_(2, 39)_ = 0.3814
Timepoint x (Placebo vs. CBD)	0.0369	^*^	Yes	*F*_(2, 39)_ = 3.595
(MR vs. no MR) x (Placebo vs. CBD)	0.5306	Ns	No	*F*_(1, 21)_ = 0.4066
Timepoint x (MR vs. no MR) x (Placebo vs. CBD)	0.9889	Ns	No	*F*_(2, 39)_ = 0.01118

The ^*^, ^**^, ^***^, and ^****^ indicates the value of *p* < 0.05, *p* < 0.01, *p* < 0.001, and *p* < 0.0001 respectively.

The mean change in the QoLI-31 score was 1.92 (2.53) and −0.18 (3.02) at 4 weeks and −0.66 (1.84) and −9.80 (2.36) at 8 weeks for placebo and CBD groups, respectively. The effect of timepoint x receiving CBD or placebo (interaction) was significant (*p* = 0.016). *Post-hoc* analysis revealed that the QoLI-31 score was significantly different between CBD and placebo groups at 8 weeks [mean diff. −8.564, 95% CI (−17.04 to −0.08956)]. Week 4 compared to week 8 change in QoL was significant for the CBD group [mean diff. −9.324, 95% CI (−16.61 to −2.040), *p* = 0.011] but not the placebo group (*p* > 0.05) (Table 7).

**Table 7:** Quality of life changes from the baseline at 4 and 8 weeks.

**Fixed effects (type III)**	***P*-value**	***P*-value summary**	**Significant?**	***F*_(DFn, DFd)_**
Timepoint	0.0639	Ns	no	*F*_(1, 20)_ = 3.849
Receiving CBD or placebo	0.2818	Ns	no	*F*_(1, 23)_ = 1.215
Timepoint x receiving CBD or placebo	0.0162	^*^	Yes	*F*_(1, 20)_ = 6.895
***Post-hoc*** **tests**				
**Multiple comparisons test**	**Adjusted** ***P*****-value**	**Significance**	**Mean difference**	**95.00% CI of diff**.
**(i) Week 4 change—week 8 change**				
Placebo	0.8602	Ns	1.350	−5.244 to 7.943
Cannabidiol	0.0114	^*^	−9.324	−16.61 to −2.040
**(ii) Placebo—cannabidiol**				
Week 4 change	0.7940	Ns	2.110	−5.927 to 10.15
Week 8 change	0.0472	^*^	−8.564	−17.04 to −0.08956

The ^*^, ^**^, ^***^, and ^****^ indicates the value of *p* < 0.05, *p* < 0.01, *p* < 0.001, and *p* < 0.0001 respectively.

**Figure 4:**
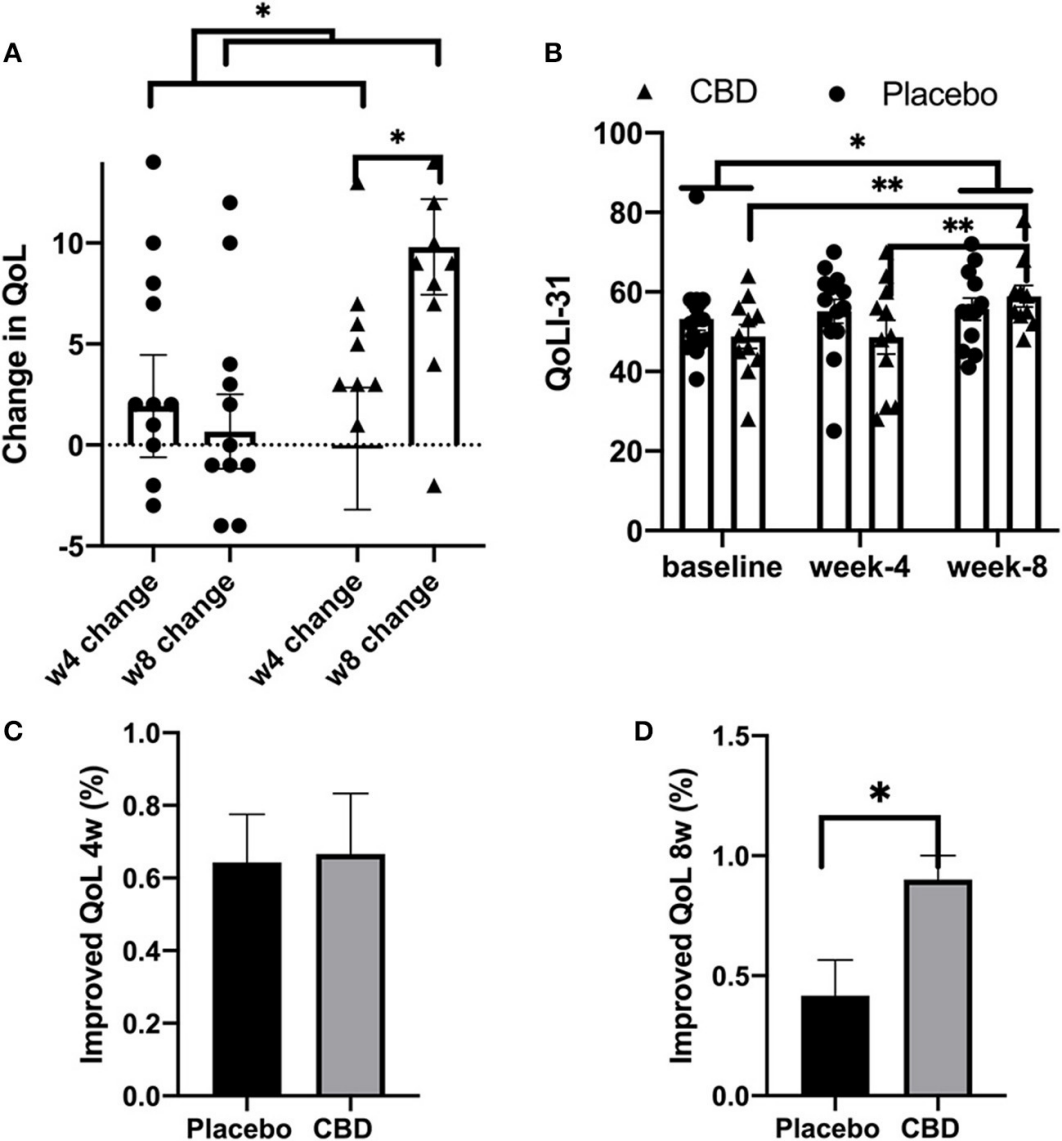
**(A–D)** Quality of life analysis. The ^*^, ^**^, ^***^, and ^****^ indicates the value of *p* < 0.05, *p* < 0.01, *p* < 0.001, and *p* < 0.0001 respectively.

Improvement in QoL was assessed as a binary parameter (improved/not improved). The difference in the percentage of patients with improved QoL was significant between the CBD and placebo patients at 8 weeks [RR: 2.160, 95% CI (1.148 to 4.741), *p* = 0.018] but not at 4 weeks (*p* = 0.653). Finally, data point out that CBD can effectively enhance QoL, particularly during longer follow-up periods (Figures 4A–D).

### Correlation analysis

**Figure 5:**
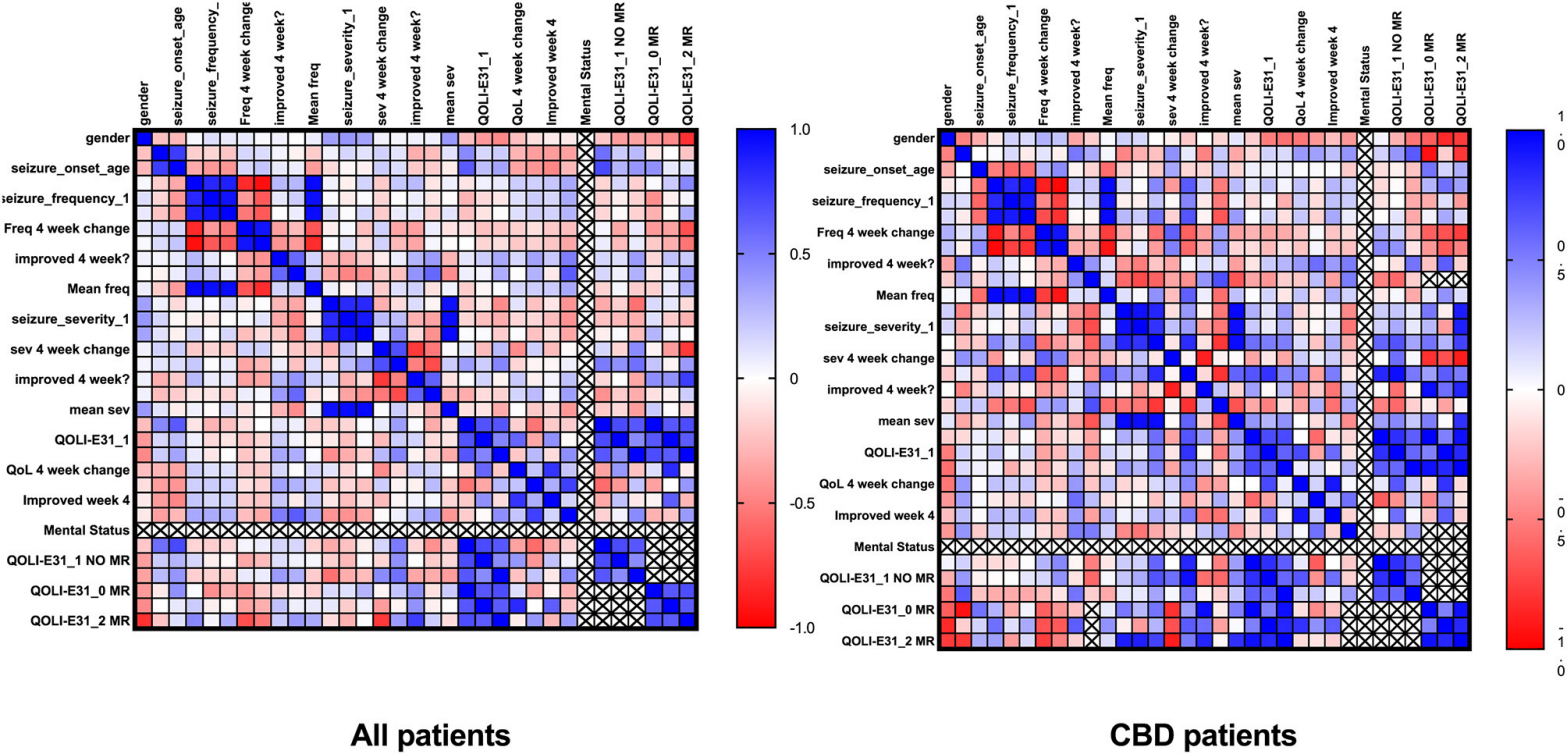
Correlation of clinical data.

We performed a correlation analysis between the study parameters for all patients. Change in seizure frequency at 4-week [*r* = −0.832, 95% CI (−0.920 to −0.661), *p* = 0.000] and 8-week [*r* = −0.938, 95% CI (−0.972 to −0.868), *p* = 0.000] timepoints were negatively correlated with baseline seizure frequency. A positive result for QoL improvement was associated with a positive result for seizure frequency reduction [*r* = 0.638, 95% CI (0.296 to 0.835), *p* = 0.001]; however, limiting the correlation analysis to cases receiving CBD indicated that QoL improvement was not associated with seizure features such as severity and frequency (*p* > 0.05). These results are plotted in Figure 5.

### Regression analysis

**Figure 6:**
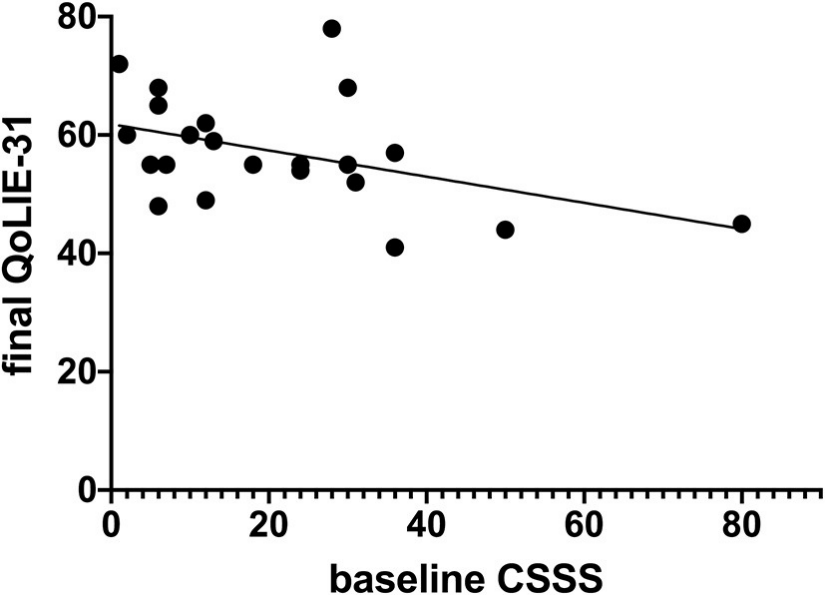
Regression analysis of seizure severity and quality of life.

In the present study, a higher seizure severity score at the baseline was a predictor of reduced QoL at the study endpoint [8 weeks; *p* = 0.036, −0.221, 95% CI (−0.427 to −0.0157), *R*^2^ = 0.201; Figure 6]. However, after restricting the analysis to the CBD group, this was not significant. Indeed, most regression models with seizure parameters as covariates failed to predict the final QoL (8 weeks).

### Adverse drug events

We studied the adverse events during the study period. The recorded side effects were sleepiness, headache, anxiety, nausea, constipation, dizziness, tremor, irritability, change in heart rate, skin problems, urinary retention, blurred vision, change in appetite, fatigue, diarrhea, dry mouth, drooling, hypotension or hypertension, dysphagia, sore throat or tongue, abdominal pain, and cough. Results showed no significant difference between each of the adverse events and the CBD and placebo groups (*p* > 0.05, for all recorded parameters).

## Discussion

Herbals and their extracts are used in various disorders (27–30). CBD is an herbal extract that has been proposed for epilepsy in different preparations. In 2018, the FDA verified the prescription of CBD-based Epidiolex for two critical types of epilepsy in pediatrics (Lennox–Gastaut and Dravet syndromes) (31). However, the utility of CBD in other treatment resistance-associated seizures is less studied. The present study extends the possible utility of CBD to the frontal subtype of treatment-resistant epilepsy.

We provided preliminary data on the efficacy and safety of CBD in this subpopulation of treatment-resistant epilepsy patients. We found an improved QoL which was not associated with alterations in seizure features. Regression analysis failed to introduce seizure parameters as predictors of QoL. This indicates that further study is recommended to determine the source of QoL improvement. Correlation analyses also confirmed the lack of association between QoL and seizure severity/frequency in the CBD group. In the interpretation of correlation data, higher baseline frequency was associated with more reduction in seizure frequency. Such correlations deserve to be explored better in the future to establish clear causal relationships. In other conditions, CBD shows diverse actions, such as improving QoL in neurodegenerative disorders and social anxiety (32) and reducing QoL in depression (33). Moreover, seizure severity is an important aspect of epilepsy. The relationship between seizure severity and QoL in epilepsy has been investigated by Harden et al. in a female population. The authors found that some domains of the QOLIE-31 correlated significantly with epilepsy severity: Seizure Worry (*r* = −0.265, *p* = 0.004) and social activity (*r* = −0.280, *p* = 0.002) (34). Similarly, in our study, seizure severity at the baseline was a predictor of QoL at the study endpoint [8 weeks; *p* = 0.036, −0.221, 95% CI (−0.427 to −0.0157), *R*^2^ = 0.201]. These findings indicate that severe and damaging seizures contribute to QoL parameters in patients with intractable seizures.

Moreover, reduced frequency of seizure was achieved with add-on CBD therapy but not routine antiseizure therapy. Earlier animal studies found that CBD diminished seizure frequency and related behavioral comorbidities in various epileptogenesis models (11). A recent randomized clinical trial found a reduction in seizure frequency in treatment-resistant epilepsy-related Lennox–Gastaut syndrome by CBD (35).

A case report article described a 10-year-old girl with refractory epilepsy and left frontal dysplasia with onset before 1 year of age. Therapy by phenytoin, topiramate, carbamazepine, lamotrigine, primidone, levetiracetam, and clobazam could not induce seizure remission. Next, she used CBD-enriched extract as an add-on therapy to antiseizure medication. This combinational treatment reduced seizures and improved general neurobehavior, speech, comprehension, and attention (36). In line with these results, we tested the effectiveness and safety of highly pure CBD in liposomal form with a significantly increased absorbance compared to previous pharmacological studies.

While we found treatment with CBD to be safe, polypharmacy with classical antiseizure medications to manage treatment-resistant seizures is often linked to serious adverse events such as sedation, somnolence, and cognitive deficits. This is commonly observed in pediatrics with specific subtypes of debilitating seizures, such as Lennox–Gastaut, Doose, and Dravet syndromes, in which affected children could be at an elevated mortality risk (37). Preclinical research for the optimal antiseizure medications has been directed toward drugs that mediate solely a single neuropathology in epileptogenesis. For instance, studies mostly focused on diminishing hyperexcitation and enhancing suppression through influencing ion channels or neural transmission. This empirical method does not utilize the multimodal intracellular elements which act as robust targets for the relief of clinical epilepsy. Interestingly, CBD is a prime candidate due to its versatile effects on the brain. CBD has the potential to attenuate inflammation (38), protect against neurodegeneration (39), stabilize neuron formation (40), and prevent oxidative stress (41).

Pharmacokinetic and pharmacodynamic reactions occur between CBD and antiseizure medications (22, 42, 43). Particularly, the cytochrome p450 system has been implicated in pharmacokinetic interactions, although not exclusively. Phenobarbital which was used by our patients is a CYP2C8/9 substrate that CBD suppresses. Phenobarbital induces both CYP3A4/CYP2C19 and could, therefore, reduce CBD (44). An animal study showed no therapeutic interaction between CBD and phenobarbital (45), and conflicting prospective evidence exists in the literature. Notably, in a study by Socala et al., CBD attenuated the antiseizure actions of levetiracetam, and this reaction is pharmacodynamic as no alterations in serum and cerebral levels of either levetiracetam or CBD were detected. Authors also found that CBD did not influence the anticonvulsant actions of lacosamide, and pharmacokinetic reactions between these two drugs cannot be dismissed as CBD enhanced the cerebral levels of lacosamide (46). However, as evidenced by RCTs, the pharmacokinetic interactions may not be significant. The recent comprehensive evaluation of evidence regarding CBD/antiseizure medication interactions mentions no significant interactions for CBD carbamazepine, clonazepam, lamotrigine, midazolam, and phenytoin (22, 42). Overall, we excluded drugs that strongly interact with CBD. The antiseizure medications in the present study may not strongly interact with CBD, yet this needs further study in future research due to discrepancies in the literature.

There were several strengths compared to previous experiments. We used CBD for the frontal subtype of treatment-resistant epilepsy, as opposed to using a mixed population of refractory epilepsy cases. Outcomes were assessed at multiple timepoints. As well, a next-generation preparation of CBD was used. The present study has several limitations. We recruited only a small number of patients. The study protocol was designed with a 14-week follow-up, yet the follow-up of patients was only feasible for 8 weeks due to problems such as the Coronavirus disease-2019 (COVID-19) pandemic. Although we faced minimal missing data, especially for seizure parameters, it was still a limitation. Another possible limitation was a lack of control for differences in antiseizure medications used in different patients. We cannot also entirely dismiss the effects of CBD impurities; however, CBD preparation in this study was highly pure at 99.95%. We suggest longitudinal controlled trials with longer follow-up periods and a larger sample size. CBD has been experimented with in NMDA-mediated seizures and found to affect opioid receptors (47, 48) and reduce inflammation through inflammasomes, major components of inflammation (49). Inflammation is a major feature of many disorders, such as epilepsy (49–51). Researchers could include such molecular implications in future *in silico* preclinical and clinical studies. Bioinformatics analyses could facilitate the exploration of molecular pathways and help determine immune-mediating aspects of therapies. These could as well be incorporated into future epilepsy studies (52–56).

Moreover, researchers could experiment with whether CBD in epileptic disorders is able to improve (executive functions and social cognition) other than the quality of life and if there is a relationship between such parameters. Indeed, research on subjects in the developmental age has shown that chronic neurological disorders such as epilepsy may be linked to difficulties in social cognition abilities and that these difficulties may be related to a deficit in executive functions, which are then essential aspects for adaptive functioning and good quality of life (57). The results on adult patients could also be corroborated in these scenarios.

Recent evidence suggests the presence of any genetic abnormalities. Polymorphisms of the SCN1A gene could play a role in the response to antiseizure medications in patients with drug-resistant epilepsy in developmental age, with key implications for clinical practice. Margari et al. showed a significant link between multiple intronic SCN1A gene polymorphisms and drug-resistant epilepsy in pediatric patients (58). We encourage further studies to focus on adults with drug-resistant epilepsy.

## Conclusion

The present study provides good-quality evidence in the form of a properly blinded and a control randomized trial of CBD for frontal drug-resistant epilepsy. CBD could reduce seizure frequency and improve QoL. Moreover, the use of CBD as an add-on therapy was found to be safe. Research on other refractory epilepsy subtypes is encouraged. Future studies should perform longer follow-ups with more effective preparations of CBD.

## Data availability statement

The raw data supporting the conclusions of this article will be made available by the corresponding author on reasonable request.

## Ethics statement

The studies involving human participants were reviewed and approved by Tehran University of Medical Sciences, code: IR.TUMS.MEDICINE.REC.1400.130. The patients/participants provided their written informed consent to participate in this study.

## Author contributions

SE, KS, TA, NR, VA, SR, and AT conceptualized the study and helped with drafting the manuscript. KS performed all statistical analyses, prepared figures, and prepared the final manuscript. SE helped with statistical analysis. SE, AT, and KS collected and evaluated patients' data. AT treated patients and supervised the study. All authors contributed to the article and approved the submitted version.
